# Impact of the phase transition of halide perovskite on the performance of ZnO ultraviolet photoconductor

**DOI:** 10.1039/d6ra08487g

**Published:** 2026-10-19

**Authors:** Femi Igbari, Muhammad Bilal, Zhe Li

**Affiliations:** a School of Engineering and Materials Science, Queen Mary University of London Mile End Road London E1 4NS UK o.igbari@qmul.ac.uk zhe.li@qmul.ac.uk

## Abstract

Despite the limitations associated with the delta-phase perovskite (δ-PVK), its potential to enhance device performance and stability has recently attracted increasing interest. In this work, we investigate the influence of these properties on the performance of ZnO-based ultraviolet (UV) photoconductors by surface-modifying the ZnO active layer with a δ-PVK layer derived from the phase transition of alpha-phase triple-cation mixed-halide perovskite, (Cs_0.05_FA_0.81_MA_0.14_)Pb(I_0.85_Br_0.15_)_3_ (α-PVK), *via* solution processing. The resulting ZnO/δ-PVK UV photoconductor exhibits enhanced performance, which is attributed to effective interfacial charge transfer from δ-PVK to ZnO, accompanied by an enhanced photogating effect. Compared with the ZnO device, the ZnO/δ-PVK device exhibits a photocurrent (*I*_photo_) *ca.* 3 times higher, while being *ca.* 2 orders of magnitude higher than that of the ZnO/α-PVK device. Accordingly, the ZnO/δ-PVK device achieves the highest *I*_on_/*I*_off_ ratio (344.81), photoresponsivity (*R* = 2.0 A W^−1^), photodetectivity (D* = 3.06 × 10^11^ Jones) and external quantum efficiency (EQE = 680%) under 365 nm UV irradiation at a power density (*P*) of 23 mW cm^−2^ and a bias voltage of 1 V. These findings highlight the potential of the previously underexplored δ-PVK phase for enhancing the performance of optoelectronic devices.

## Abstract 2

Enhanced charge transfer and photogating effect enabled a ZnO/δ-PVK heterostructure UV photoconductor to outperform its ZnO single-layer counterpart in UV sensing.

## Graphical abstract

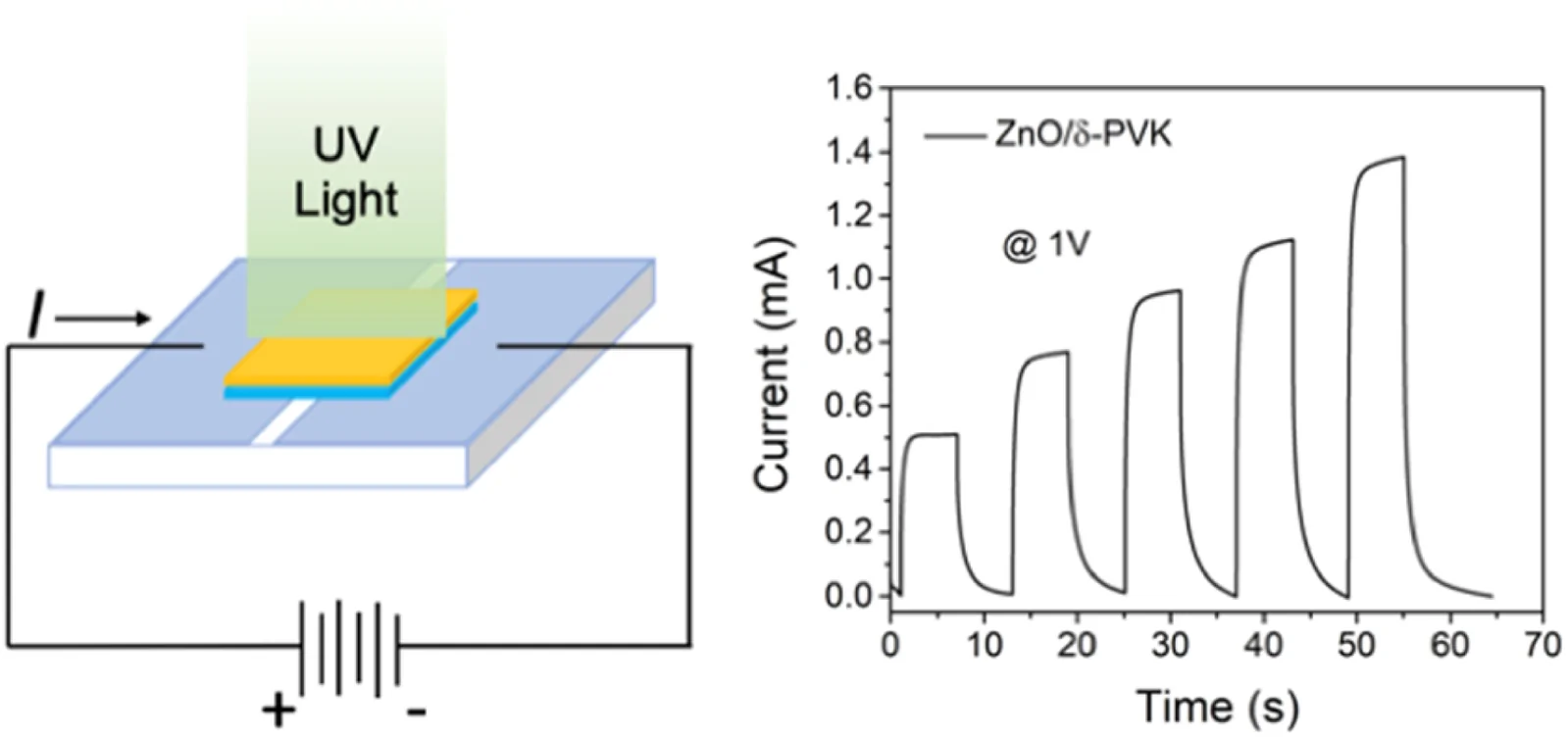

## 1. Introduction

Hybrid organic–inorganic halide perovskite (PVK) thin films have emerged as promising active materials for optoelectronic devices due to their defect tolerance, tunable band structure, long carrier diffusion length, high mobility, strong light absorption and ease of processing.^1–11^ These properties have enabled remarkable progress in photovoltaic devices, with power conversion efficiencies exceeding 27% and have also supported applications in photosensors with high responsivity (*R*), detectivity (D*) and external quantum efficiency (EQE).^2–9,11–14^ Studies have demonstrated flexible, highly sensitive PVK photodetectors, performance enhancement through interface passivation, wavelength-selective narrowband detection and improved device characteristics *via* metal oxide/PVK heterostructures such as ZnO/CsPbBr_3_.^13–15^

Despite these advantages, practical deployment of halide PVKs is hindered by toxicity and poor stability.^16,17^ Their preferred α-phase is thermodynamically unstable and can readily transform into the δ-phase under heat or humidity.^18^ Although δ-phase PVKs possess inferior visible-light optoelectronic properties due to wider band gaps, stronger electron–phonon coupling and lower carrier mobility,^19–21^ they offer superior stability and are well suited for higher-energy applications such as UV and X-ray detection.^22–25^

Expanding on our preliminary results previously presented under separate measurement conditions,^26^ we further explore the effect of PVK phase transition on the performance of ZnO UV photoconductor. To-date ZnO/PVK heterostructure photosensing works have focused mainly on α-PVK.^15,23^ Moreover, ZnO/δ-PVK heterostructure which can enhance UV-sensing by combining ZnO UV sensitivity with wide bandgap δ-PVK has not been reported. Hence, our device performance significantly improved when the PVK layer is in the δ-phase. The enhanced performance can be attributed to the δ-PVK exhibiting; (i) better stability against UV light irradiation compared to α-PVK, (ii) effective UV photon absorption and electron–hole pair formation due to suitable band gap, (iii) favorable energy level alignment with ZnO which overcomes interface built-in potential, facilitating electron injection into the ZnO conduction band, (iv) enhanced interface-induced photogating effect which facilitates spatial charge separation and accumulation, suppressing recombination.

As an extension of our previous report,^26^ additional data and analyses are presented here to further substantiate these conclusions. Comparing the results obtained in this work with those from our previous report provides valuable insight into the dependence of device performance metrics on measurement conditions, indicating that these metrics should not be regarded as intrinsic, single-valued properties of the devices. Moreover, despite variations in their absolute values, the devices exhibit consistent performance trends under both measurement conditions. These findings highlight the importance of clearly specifying measurement conditions when reporting and comparing the performance of perovskite-based photoconductors.

## 2. Results and discussion

As shown in Fig. 1, a device achieving back-contact architecture and exhibiting effective light area of 0.03 cm^2^ was fabricated on patterned FTO substrate. The active layer was grown across the area to achieve a ZnO-, ZnO/α-PVK- and ZnO/δ-PVK-based device at each stage of the process. To confirm phase formation and α to δ phase transition, XRD patterns of the active layer were obtained as presented in Fig. 2a. Although small residual PbI_2_ was detected in both films, the PVK layer before and after phase transition is hereafter denoted as α-PVK and δ-PVK respectively according to their predominant perovskite phases. Inset is an illustration of the transition from α-phase corner-sharing to δ-phase edge-sharing octahedra. The intense peaks at 31.9, 34.5 and 36.3 on the black pattern show the formation of pure ZnO wurtzite phase while the intense peaks at 14.5 on the red pattern and 11.7 on the blue pattern confirm the PVK phase transition from α to δ.

**Fig. 1:**
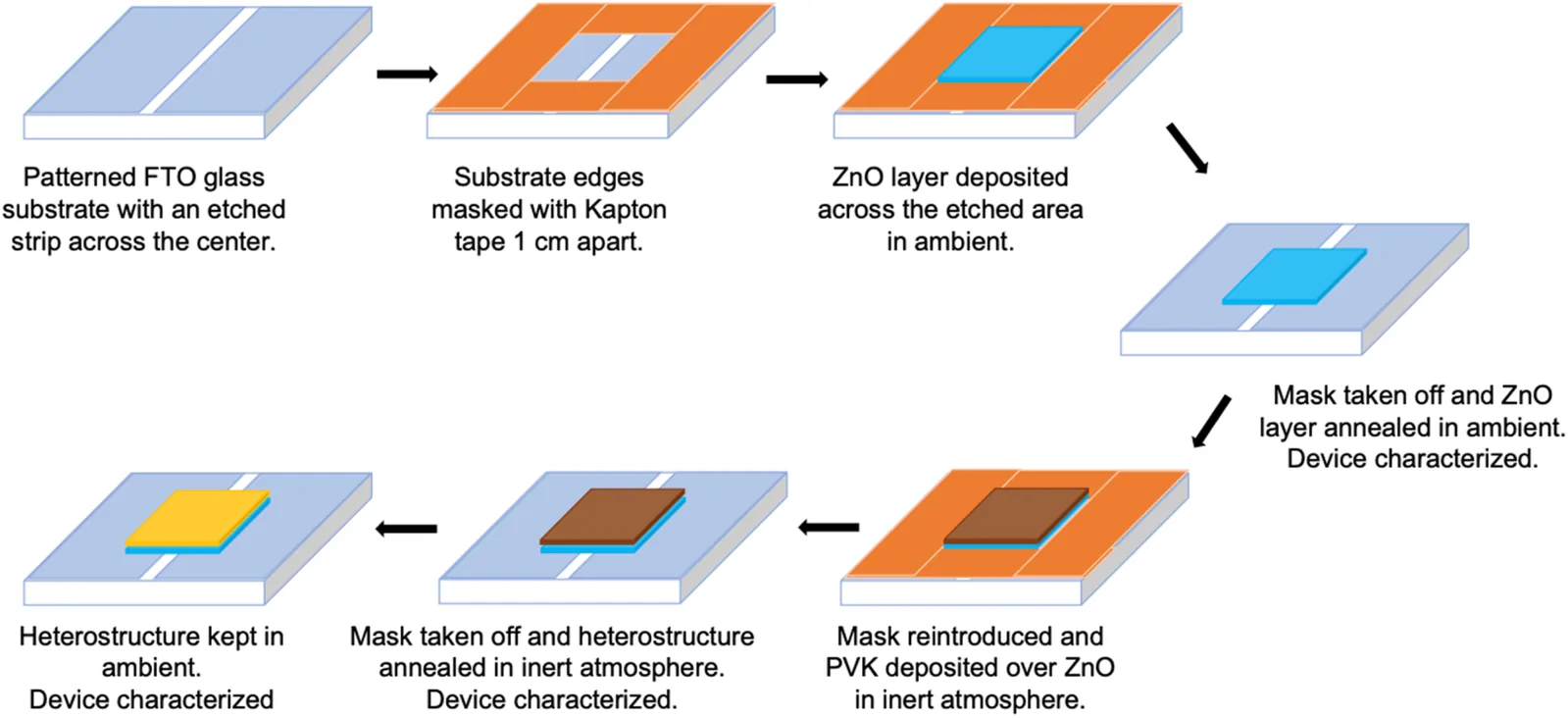
Schematic description of the device fabrication process.

**Fig. 2:**
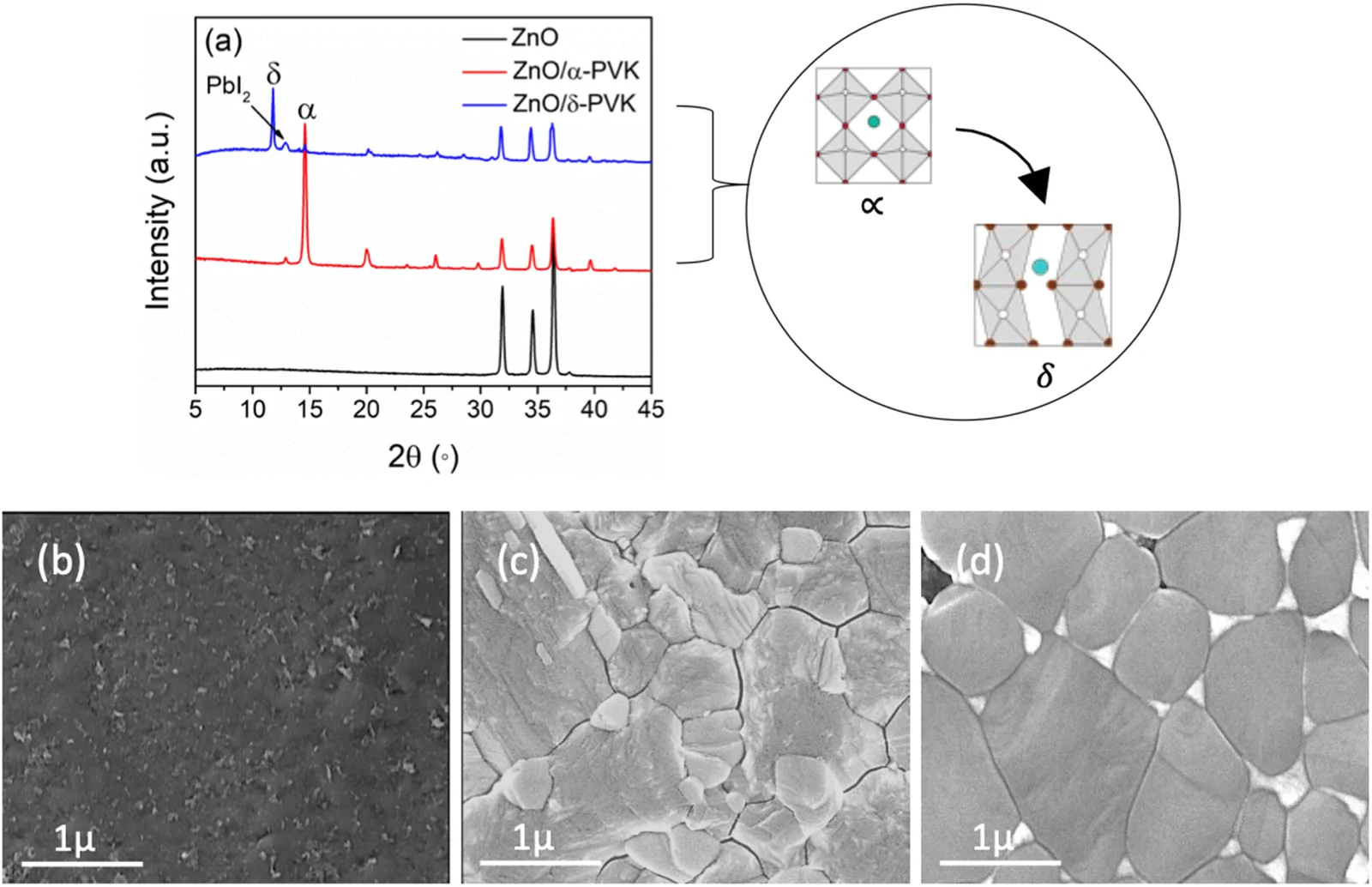
(a) XRD patterns of pristine ZnO compact layer, ZnO/α-PVK and ZnO/δ-PVK heterostructures. Inset is an illustration of the attendant lattice change. SEM images of the (b) ZnO, (c) α-PVK and (d) δ-PVK surfaces.

Notably, the phase transition product denoted as δ-PVK is characterized by an intense δ-phase peak at 11.7, while the much weaker peaks at 12.6 and 14.5 correspond to residual PbI_2_ and α-PVK, respectively. The weak α-PVK signal persists due to the relatively good stability of triple cation PVKs under ambient conditions, even with extended exposure.^27–29^ Moreover, such exposure also leads to the appearance of PbI_2_ peaks in the XRD pattern, arising from the loss of organic components and a resulting shift in sample stoichiometry, which leaves PbI_2_ in excess.^28^ The basic nature of the ZnO underlayer, which thermodynamically promotes interface-driven degradation and defect formation during ambient ageing^30–35^ also hinders the formation of a clean δ-phase. Table S1 shows the ZnO, α-PVK and δ-PVK films thicknesses as 486 ± 6, 254 ± 7.2 and 242 ± 10 nm, respectively, revealing substantially thinner α-PVK and δ-PVK layers compared to the underlying ZnO layer. This enhances the influence of the basic surface of ZnO on the adjacent PVK layer and contributes to the realization of the α-phase at a lower annealing temperature while accelerating the subsequent transformation to the δ-phase under ambient conditions (see Experimental section in SI) compared to the PVK-only sample where such interactions are absent. Similar ZnO-induced interfacial instability has been previously reported.^36,37^ It is evident in the XRD patterns of the heterostructure in Fig. S1 that prolonged heating at elevated temperatures (*i.e.*, 40 °C, 60 °C and 80 °C) in ambient resulted in further degradation and intensification of the PbI_2_ peak. The underlayer also limits the coverage by the polycrystalline PVK top layer, causing wide grain boundaries and voids and accounting for the appearance of ZnO peaks on the ZnO/α-PVK and ZnO/δ-PVK heterostructures XRD patterns. Such surface imperfection intensifies with prolonged heating as evident in the shrinkage of the gains and widening of the void areas on the SEM images in Fig. S2a–c. SEM images of the devices are presented in Fig. 2b–d. The dense and compact ZnO layer provides complete coverage of the etched area. However, regions of wide grain boundaries and voids extending down to the ZnO layer are present on the PVK layer, which further explains the presence of ZnO peaks in the XRD patterns of the heterostructures. Changes in surface morphology between the α- and δ-PVK coupled with the appearance of segregated areas on the δ-PVK image further support the phase transition.

Moreover, good interfacial contact between the heterostructures and the FTO electrode ensures the formation of ohmic junction. This is demonstrated by the linear plots of the *I*–*V* characteristics of the devices going through the origin, measured under both dark and illuminated conditions across a bias voltage range of −1 V to +1 V, at a power density (*P*) of 23 mW cm^−2^ and a wavelength of 365 nm, as shown in Fig. 3a–c. Corresponding logarithmic plots are presented in Fig. S3a–c. In the heterostructure devices, ZnO acts as both UV absorbing active layer and electron transporting layer. At 1 V bias, the photocurrent achieved by the ZnO based device dropped by 1.4 orders of magnitude when the α-PVK top layer was introduced to form a ZnO/α-PVK heterostructure. Moreover, a *ca.* 3-fold increase in the photocurrent was achieved after the phase transition of the α-PVK to form a ZnO/δ-PVK heterostructure.

**Fig. 3:**
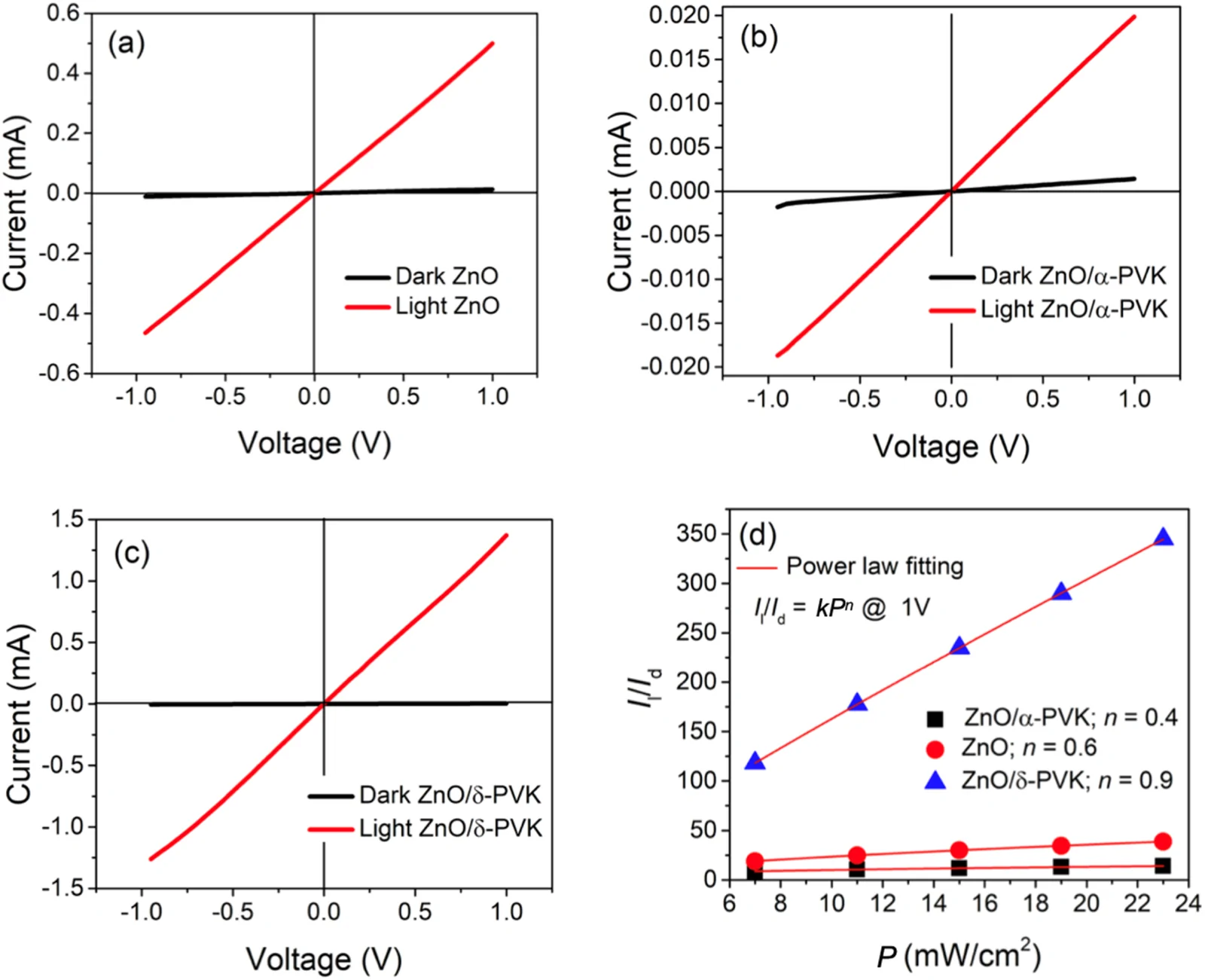
*I*–*V* characteristics of the devices based on (a) ZnO, (b) ZnO/α-PVK and (c) ZnO/δ-PVK in the dark and under UV irradiation. (d) Power law plots for devices.

Similar trend was observed for the extracted on/off current ratio (*I*_l_/*I*_d_) which are 38.70, 14.04 and 344.81 for ZnO, ZnO/α-PVK and ZnO/δ-PVK-based devices respectively. It can be seen that the introduction of δ-PVK top layer increases the *I*_l_/*I*_d_ of the ZnO based device by *ca.* 10-fold while α-PVK decreases it by *ca.* 3-fold. This is attributed to the lower intrinsic conductivity and charge mobility of the δ-PVK compared to the α-PVK which can achieve lower dark current and suppression of leakage current in the ZnO layer. Fig. S4a–c shows the *I*–*V* characteristics of the devices with increasing *P* (7–23 mW cm^−2^) at the same test conditions. The photocurrent increases steadily with increasing photon intensity, confirming their photosensitivity. These increases indicate that *I*_l_/*I*_d_ follows a power law which is dependent on the light intensity. Hence, *I*_l_/*I*_d_ can be evaluated as a function of *P* at constant test conditions using the power law equation (*I*_l_/*I*_d_ = *kP*^*n*^).^38–42^ Where *I*_l_ is the photocurrent, *I*_d_ is the dark current, *k* is a constant and *n* is the power law index evaluated from the fitted curve of *I*_l_/*I*_d_*vs. P*. As shown in Fig. 3d, *n* for the ZnO/δ-PVK-based device (*n* = 0.9) is closest to linearity than those of ZnO- and ZnO/α-PVK-based devices (*i.e. n* = 0.6 and 0.4) respectively, demonstrating that the ZnO/δ-PVK-based device achieves the highest UV sensitivity.

Furthermore, chronoamperometry was done to investigate the photoswitching properties of the devices with increasing *P*. The device operation is schematically illustrated in Fig. 4a. Fig. 4b–d reveal that the photocurrent increased with increasing *P* similar to the *I*–*V* characteristics in Fig. S4a–c due to the increase in photon intensity. The on/off speeds were sampled from the time dependent normalized response at 23 mW cm^−2^ as presented in Fig. S5a–c. The ZnO/δ-PVK-based device yielded rise time (*T*_r_) and fall time (*T*_f_) of 0.6 s and 1.3 s respectively while the ZnO- and ZnO/α-PVK-based devices yielded *T*_r_/*T*_f_ of 1.1 s/2.4 s and 1.7 s/3.2 s respectively. They show that, the ZnO-based device exhibits the shortest *T*_r_/*T*_f_ with the introduction of a δ-PVK top layer. Changes in the figures of merit *i.e. R*, D*, EQE of the device before and after the introduction of PVK top layer including PVK phase transition were extracted from Fig. 3a–c using the equations: *R* = (*I*_photo_ − *I*_dark_)/(*PA*); 
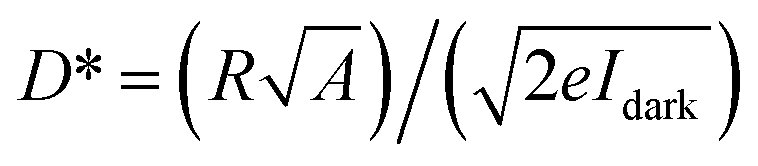
; EQE (%) = (*Rhc*/*eλ*) × 100,^39–41^ respectively. Where *A* is effective area, *e* is electron charge, *h* is Planck's constant, *c* is speed of light and λ is wavelength. Table 1 shows that our device achieved the highest performance metrics when the ZnO/δ-PVK heterostructure is formed. The highest values obtained for *R*, D* and EQE also compare well with reported values from ZnO and ZnO/PVK devices as presented in Table S2.

**Fig. 4:**
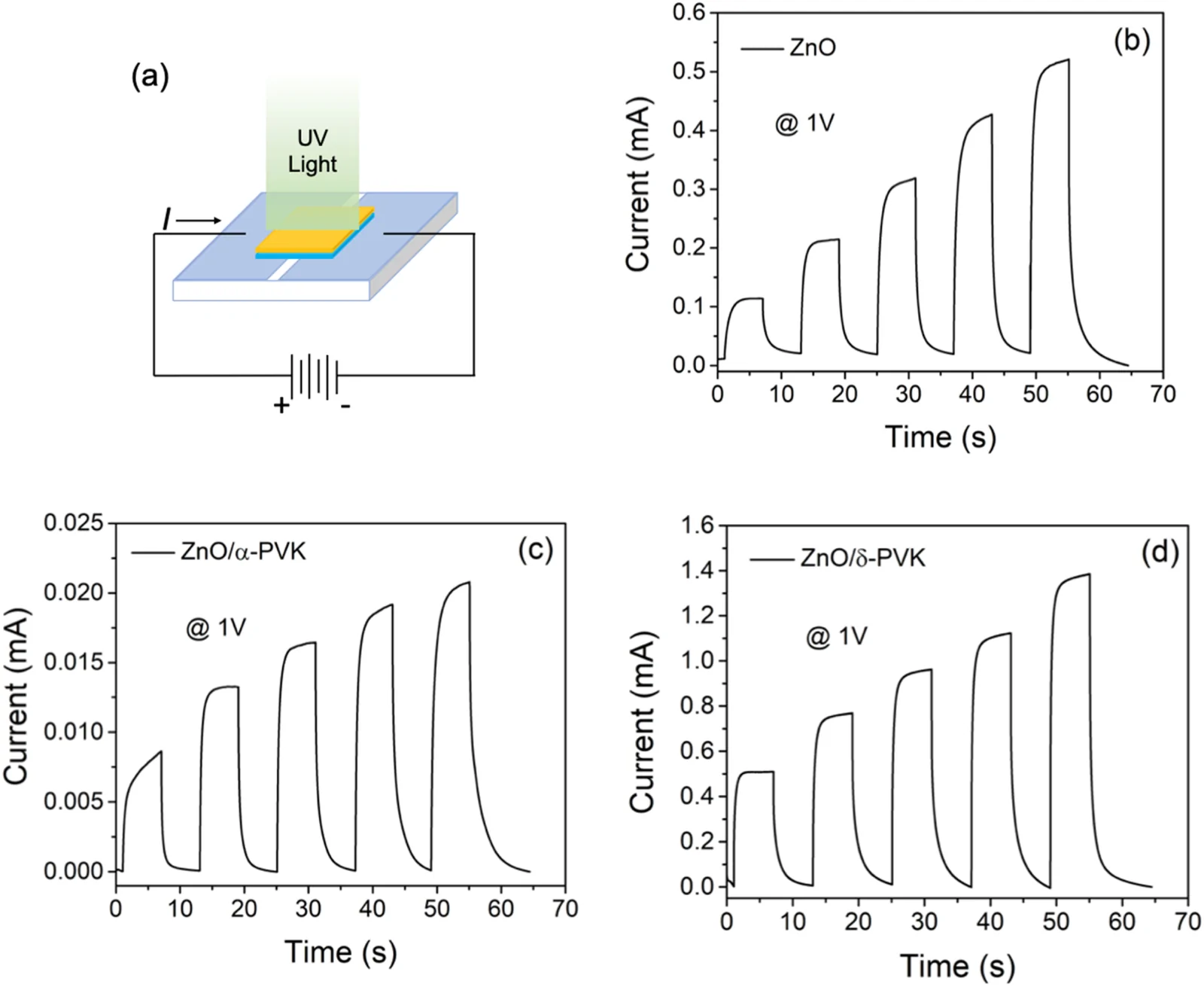
(a) Schematic illustration of the photoconductor irradiated with UV light. Photoswitching property of the devices based on (b) ZnO, (c) ZnO/α-PVK and (d) ZnO/δ-PVK at 7, 11, 15, 19 and 23 mW cm^−2^*P*, 1 V bias and 365 nm wavelength of UV light.

**Table 1:** Device parameters

Active layer	Bias (V)	*λ* (nm)	*T*_r_/*T*_f_ (s/s)	*I*_l_/*I*_d_	*R* (A W^−1^)	*D* (Jones)	EQE (%)
ZnO	1	365	1.1/2.4	38.70	0.7	5.96 × 10^10^	238
ZnO/α-PVK	1	365	1.7/3.2	14.04	0.03	7.70 × 10^9^	10
ZnO/δ-PVK	1	365	0.6/1.3	344.81	2.0	3.06 × 10^11^	680

Furthermore, Fig. S6 presents box plots of *R*, D* and EQE obtained from ten independently fabricated devices. The ZnO/δ-PVK devices exhibit the highest median *R*, D* and EQE, followed by the ZnO devices while the ZnO/α-PVK devices show the lowest values. The narrow interquartile ranges and absence of pronounced outliers indicate good device-to-device reproducibility for all three device types. The relatively small spread observed for the ZnO/δ-PVK devices suggests that the enhanced performance is consistently achieved across multiple samples rather than arising from a single exceptional device. Therefore, compared to ZnO devices, incorporation of the α-PVK decreases the median *R* by approximately one order of magnitude, while the δ-PVK enhances it by nearly threefold.

Similar trends are observed for D* and EQE, indicating the superiority and reproducibility of the ZnO/δ-PVK device performance.

Also, the extended UV irradiation test at 365 nm wavelength, 23 mW cm^−2^ power density and 1 V bias presented in Fig. 5 shows that although the photocurrent of the ZnO/δ-PVK device is significantly higher, both ZnO and ZnO/δ-PVK devices exhibit nearly constant photocurrent throughout the measurement period, indicating excellent photostability and stable charge transport under continuous UV illumination. In contrast, the ZnO/α-PVK device shows significantly low and an immediate decay in photocurrent, suggesting the occurrence of UV light-induced degradation processes that reduce carrier collection efficiency over time. This observation is also supported with XRD results (Fig. S7) showing an appearance of a more defined PbI_2_ peak and a reduced intensity of the α peak for the ZnO/α-PVK heterostructure, while the XRD patterns of the ZnO and ZnO/δ-PVK heterostructure remain largely unchanged compared to the XRD patterns (Fig. 2a) obtained before testing. This behaviour is attributed to the poor stability of α-PVK under prolonged UV irradiation. In addition, the chemical incompatibility between ZnO and α-phase halide perovskites can accelerate interfacial degradation through reactions at the ZnO/α-PVK interface, further suppressing charge extraction. The stable response observed for the ZnO/δ-PVK device indicates that the δ-PVK is significantly more resistant to this degradation, owing to its higher structural and environmental stability, thereby maintaining a consistent photocurrent during continuous operation.

**Fig. 5:**
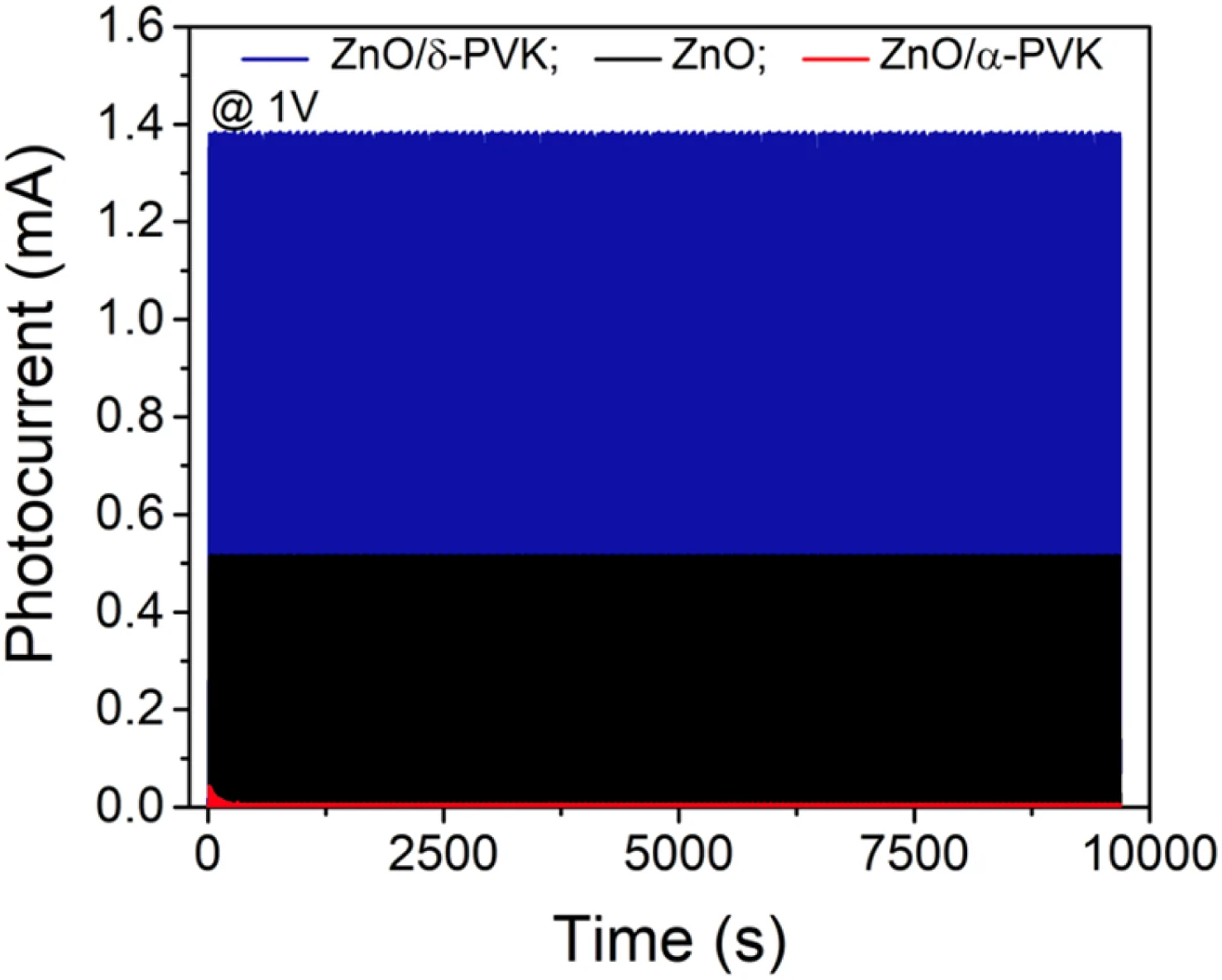
Photocurrent stability measurements taken at 1 V, 365 nm and 23 mW cm^−2^ light density.

To gain insight into the enhanced device performance, photophysical properties were studied. UV-vis absorption spectra of ZnO, α-PVK, δ-PVK thin films were measured and presented in Fig. 6a. The films exhibit absorption onsets at *ca.* 365 nm, 730 nm and 413 nm respectively with α-PVK and δ-PVK exhibiting the highest and lowest absorption intensities around the test wavelength (*i.e.*, 365 nm) respectively. This is attributed to the stronger electronic coupling, narrower bandgap and strongly allowed direct transition as well as higher density of states characteristics of α-PVK compared to ZnO and δ-PVK.^43–45^ The Tauc plots extracted from corresponding absorption spectra are presented as the inset of Fig. 6a. The bandgaps are estimated at 3.4 eV, 1.7 eV and 3.0 eV for the ZnO, α-PVK and δ-PVK thin films respectively, in good agreement with reported values.^15,27–30,34,46–48^ Moreover, photocurrent is not determined by photon absorption alone. It is dominated by how efficiently the material converts absorbed photons into extracted, non-recombined charge carriers under operating conditions. The ZnO based photoconductor shows the highest photocurrent when δ-PVK top layer is introduced because the resulting ZnO/δ-PVK heterostructure combines effective UV-induced charge transfer with strong interfacial separation and extremely low dark current, maximizing the photogenerated charges better than the ZnO and ZnO/α-PVK based photoconductors.

**Fig. 6:**
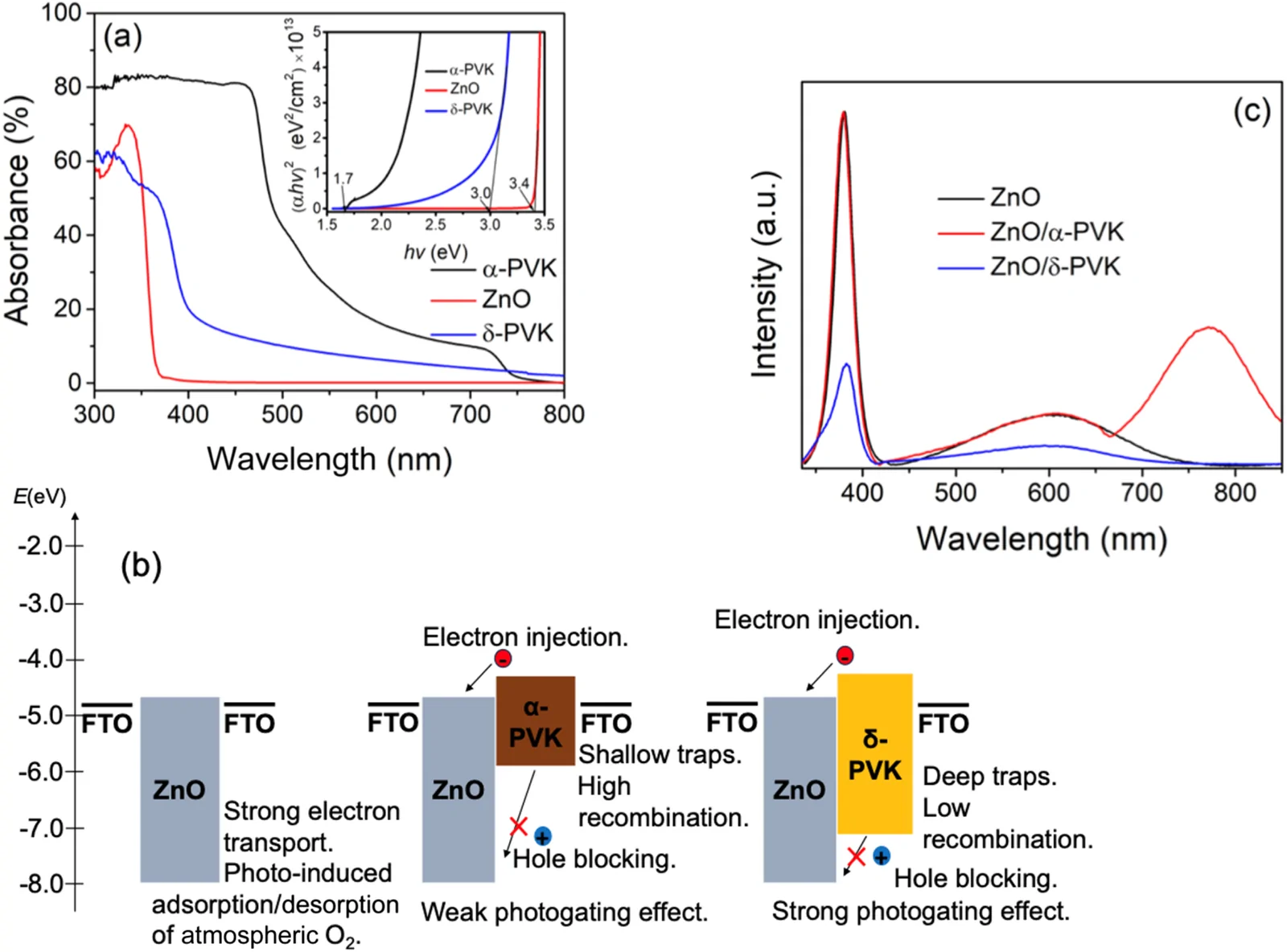
(a) UV-vis absorption spectra of ZnO, α-PVK and δ-PVK thin films. Inset are the corresponding Tauc plots. (b) Energy level alignments and operation mechanism of the devices. (c) Steady state PL spectra of the ZnO device before and after the introduction of α-PVK and δ-PVK top layers.

Fig. 6b shows the energy level diagram and operational mechanism of the ZnO-based device before and after the introduction of α- and δ-PVK. The origin of the photoresponse in the ZnO device is an active adsorption/desorption process.^38,47^ Under dark condition, atmospheric oxygen species (O^−^, O_2_^−^) are adsorbed onto the ZnO surface. The oxygen species capture free electrons, creating a depletion layer and suppressing charge carrier concentration. Moreover, under UV light irradiation, photons are absorbed and electron–hole pairs are created. The holes displace the captured electrons, effecting the desorption of the oxygen species. Both the free electrons and photogenerated electrons are excited into the conduction band. This reduces the depletion layer and enhances the photoconductivity of the ZnO layer. Notably, this process creates intrinsic oxygen-induced photogating effect^49,50^ which increases as *P* intensifies and as observed on Fig. S4a.

Moreover, the photogating effect changes with the introduction of different phases of PVK top layer. To better understand this phenomenon, ultraviolet photoelectron spectroscopy (UPS) together with the UV-vis absorption spectroscopy and inset Tauc plot results shown in Fig. 6a were employed. Fig. S8a–c shows the UPS spectra of ZnO, α-PVK and δ-PVK thin films which were obtained using a helium (He) lamp with an excitation energy of 21.22 eV. The valence band maxima (VBM) of the films are −7.98 eV, −5.81 eV and −7.10 eV respectively as presented in Table S3. By combining these values with the bandgaps derived from the Tauc plots, the corresponding conduction band minima (CBM) are determined to be −4.58 eV, −4.11 eV and −4.10 eV respectively. Therefore, the energy level alignments of the devices illustrated in Fig. 6b show that both α-PVK and δ-PVK are capable of effecting electron injection into the ZnO layer. This is evident in their higher CBM and comparable energy level offset relative to ZnO.

Notably, despite the stronger photophysical properties of α-PVK, the enhanced performance of the ZnO UV photoconductor with a δ-PVK top layer is primarily governed by interfacial effects under UV illumination. In the ZnO/α-PVK device, UV irradiation excites both ZnO and α-PVK. α-PVK generates free carriers efficiently. Electrons are injected into the ZnO layer due to favorable band alignment. However, this achieves weak interfacial photogating^51^ as a result of limited interface charge accumulation due to shallow traps and fast recombination in the α-PVK. Moreover, for the ZnO/δ-PVK based device, UV irradiation strongly excites ZnO. The wider bandgap of δ-PVK could potentially favour the formation of deep trap states, which contribute to enhanced interfacial photogating through the trapping of photogenerated charges (holes). The trapped charges create electric field which gate the ZnO channel electrostatically. It results in amplified ZnO conductivity as the ZnO electrons are modulated by its own oxygen-induced gating as well as the strong gating from trapped charges, yielding high performance metrics in the device. The shortest *T*_r_ and *T*_f_ of the ZnO/δ-PVK device (Table 1) also suggest that its response is not dominated by slow recombination or capacitive loading. Hence, the δ-PVK improved the device performance mainly by suppressing interfacial recombination as well as acting as a charge-blocking and accumulation layer. It supports the significantly higher photocurrent and superior long-term operational stability shown in Fig. 5, despite the lower absorption of δ-PVK at 365 nm compared with α-PVK as shown in Fig. 6a. It indicates that the enhanced photoresponse of ZnO/δ-PVK is not governed solely by the optical absorption of the perovskite layer, but is strongly influenced by favourable charge trapping/detrapping dynamics and interfacial photogating provided by the UV-stable δ-PVK layer.

This is further supported by the steady state PL spectra (excitation wavelength; 365 nm) of the ZnO device before and after the introduction of α-PVK and δ-PVK top layers shown in Fig. 6c. The near band and defect emission peaks are at 380 nm and 607 nm respectively. The ZnO peak positions remain unchanged regardless of the PVK phase, since there is no alteration in bandgap, composition or alloy formation of the ZnO. However, the ZnO peaks are significantly quenched upon the introduction of δ-PVK, which can be attributed to the photogating and charge separation effects suppressing emissive recombination. In contrast, when α-PVK is introduced, the ZnO peak intensities remain unaffected. An additional peak also emerges at 770 nm due to radiative recombination and strong emissive property contribution by α-PVK. Hence despite the small amount of residual PbI_2_ in the XRD patterns of both the α-PVK and δ-PVK films (Fig. 2a), which may enhance the photoresponse of both ZnO/α-PVK and ZnO/δ-PVK devices through PbI_2_-mediated bulk and interfacial defect passivation,^52^ the significantly higher photoresponse of the ZnO/δ-PVK device combines this with distinct interfacial and electronic contributions from the δ-PVK phase. In particular, the significantly higher photocurrent observed for the ZnO/δ-PVK heterostructure compared with both pristine ZnO and ZnO/α-PVK (Fig. 4b–d), together with the PL quenching upon δ-PVK incorporation (Fig. 6c), supports the occurrence of efficient interfacial charge separation and transfer rather than PL suppression through enhanced interfacial or non-radiative recombination. Furthermore, the stable photocurrent of ZnO/δ-PVK under prolonged UV irradiation, in contrast to the progressive photocurrent decay observed for ZnO/α-PVK in Fig. 5, further supports the formation of a stable photoactive interface with favourable charge trapping/detrapping dynamics in the δ-PVK-containing heterostructure, rather than the introduction of additional recombination centres. Notably, the imperfect PVK coverage revealed by the XRD and SEM measurements creates a coexistence of photogating effects arising from both the oxygen adsorption/desorption in the ZnO as well as the charge trapping in the PVK. Moreover, stronger charge trapping in the δ-PVK resulted in the superior photocurrent and overall performance metrics observed in the ZnO/δ-PVK heterostructure device.

## 3. Conclusion

In summary, the impact of the α-to-δ phase transition of PVK on the performance of ZnO UV photoconductor was systematically investigated. XRD analysis confirmed both the crystallinity of ZnO and the phase transition of PVK, while UV-vis spectroscopy, Tauc plots and UPS measurements elucidated the energy band alignments of the active layers. The superior performance of the ZnO/δ-PVK device was attributed to enhanced photogating effects, more efficient charge separation, favorable energy-level alignment, suppressed charge recombination and increased photocurrent generation. Although the present device performance remains below the state of the art for thin-film photodetectors, the ZnO/δ-PVK heterostructure highlights the potential of δ-phase perovskites, which have hitherto been largely overlooked in optoelectronic devices, beyond their well-known superior stability. By exploiting their electronic structure, favourable charge trapping/detrapping capability and interfacial charge modulation properties through photogating, the ZnO/δ-PVK heterostructure achieves enhanced performance metrics compared with the pristine ZnO and the ZnO/α-PVK heterostructure.

## Author contributions

Femi Igbari: visualization, conceptualization, methodology, investigation, writing – original draft, formal analysis, revision, editing, data curation, validation. Muhammad Bilal: methodology, investigation, formal analysis. Zhe Li: conceptualization, revision, editing, validation, supervision, funding acquisition, project administration.

## Conflicts of interest

There are no conflicts of interest to declare.

## Supplementary Material

RA-016-D6RA08487G-s001

## Data Availability

The data supporting this article have been included as part of the main manuscript and in the supplementary information (SI). Supplementary information: experimental section. See DOI: https://doi.org/10.1039/d6ra08487g.
