# Supplementary material for: Impact of the phase transition of halide perovskite on the performance of ZnO ultraviolet photoconductor
Source: RSC Adv. 2026 Oct 19;16(48):64338–45. doi: 10.1039/d6ra08487g (PMC13678671; doi:10.1039/d6ra08487g)
Supplement: Supplementary file 1 [file RA-016-D6RA08487G-s001.pdf]

Supplementary Information (SI) for RSC Advances.
This journal is © The Royal Society of Chemistry

Supporting Information

Impact of the phase transition of halide perovskite on the performance of ZnO ultraviolet photoconductor

Femi Igbari, Muhammad Bilal, Zhe Li

School of Engineering and Materials Science, Queen Mary University of London, Mile End Road, E1 4NS.

Experimental section

Materials: $(Cs_{0.05}FA_{0.81}MA_{0.14})Pb(I_{0.85}Br_{0.15})_3$ triple cation PVK ink (5mL; I301) was purchased from Ossila Ltd, 99.8% ethyl acetate (anhydrous), zinc oxide nanoparticle ink (2.5 wt. % (crystalline ZnO in 2-propanol)), 99.5% acetone, absolute ethanol, Hellmanex III were purchased from Merck Life Science UK Ltd and 15 Ω/sq patterned fluorine-doped tin oxide (FTO) glass substrates were purchased from X & X Technology, deionized water (DI H_2O) was prepared in our lab using an LWP-F5-M series water purifier.

Preparation of Thin films: 1 ml of the ZnO dispersion nanoparticles was transferred into a sample vial and was left to agitated for 1 hr on a magnetic stirrer at room temperature. Patterned FTO glass substrate was cleaned by successive sonication in diluted Hellmanex III (2 vol. % in DI H_2O), DI H_2O, acetone and ethanol for 10 min each. The substrate was blown dry with compressed N_2 gas and was further dried on a hotplate at 100 °C for 1 hr. The substrate was then subjected to UV-Ozone cleaning for 30 min. The ZnO dispersion nanoparticles were immediately deposited by spin-coating at 3000 rpm for 35 sec and annealing at 150 °C for 5 min. The spin-coating and annealing steps were repeated 10 times and the resulting ZnO layer was annealed at 400 °C for 10 min to achieve the desired ZnO layer thickness, consistency, compactness and crystallinity. Two clean substrates were moved into a N_2 gas filled glovebox where the O_2 and H_2O levels were maintained at 0.1-0.3 ppm and 0.1-0.5 ppm respectively throughout the process. PVK ink was agitated for 30 min in the glovebox and 50 μl of the ink was immediately deposited using a two-step spin-coating method involving a first step of 1000 rpm for 10 sec followed by a second step of 3000 rpm for 30 sec. 200 μl of ethyl acetate antisolvent was dynamically casted 13 sec into the second step. The films were annealed at 110 °C for 10 min to achieve □-PVK. One film was taken out of the glovebox and kept in ambient (*T*: 23-25 °C; R.H: 40-50%) for 72 hrs to induced the phase transition of □- to δ-PVK. The photophysical properties of the obtained ZnO, □-PVK and δ-PVK layers were characterized.

Fabrication of UV Photoconductor: Patterned FTO glass substrate was used. The substrate was cleaned as described above. Their edges were carefully masked with Kapton tape to create an effective light area of 0.03 cm². The substrate was then subjected to UV-Ozone cleaning for 30 min. The ZnO nanoparticles were deposited as described above to

achieve the ZnO UV photoconductor. The photosensing and photoswitching properties of the device were characterized. Thereafter, the ZnO surface was treated with UV-Ozone for 20 min and the device was transferred into a N_2 gas filled glovebox. The PVK ink was spin-coated on the ZnO layer as described above and the resulting film was annealed at 90 ^{o}C for 10 min to achieve the ZnO/□-PVK UV photoconductor. The device was taken out of the glovebox and the photosensing and photoswitching properties were immediately characterized. The device was then kept in ambient (*T*: 23-25 $^{\circ}$C; R.H: 40-50%) for 24 hrs to induced the phase transition of □- to δ-PVK and achieve the ZnO/δ-PVK UV photoconductor. The photosensing and photoswitching properties were again characterized.

Characterization of thin films and active layers: The active layer thicknesses were measured at six different locations across each sample using an Alpha-Step D-600 Stylus Profiler. The ZnO/□-PVK and ZnO/δ-PVK heterostructure thicknesses were measured and the ZnO thickness (initially measured) was subtracted from the obtained values to determine the □-PVK and δ-PVK thicknesses as shown in Table S1. The devices were illuminated from the rear through the glass substrate. The surface morphologies of the ZnO, ZnO/□-PVK and ZnO/δ-PVK layers were analyzed on a Field Emission Scanning Electron Microscope (FE-SEM; S-4700, HITACHI). Thin film XRD measurements were conducted, using an Empyrean Diffractometer (PANalytical) emitting Ni-filtered CuKa radiation from a copper sealed tube charged with 40kV voltage and 40mA current in the Bragg-Brentano geometry, to confirm the crystal phases of the layers. The diffraction patterns were obtained by step scanning within the 2θ range of 5-45° scattering angle and steps of 0.017°. Ultraviolet-visible (UV-Vis) absorption spectra were obtained on a Perkin Elma Lambda 950 UV-Vis-NIR spectrophotometer within the range of 350 nm to 800 nm. The interface charge transfer and energy level alignments between the different phases of the PVK and the ZnO were evaluated from PL peak intensities obtained using an FLS1000 Photoluminescence Spectrometer and UPS data obtained using a Thermo Scientific ESACLAB 250Xi Ultraviolet Photoelectron Spectrometer.

Characterization of UV Photoconductor:

Device geometry and measurement conditions: Using Trumpf Laser Marking System at 355nm, 0.03cm wide line was etched across the center of an FTO glass substrate to disrupt the conduction continuity of the FTO layer. The photodetector was fabricated in a back-contact architecture with the active layer deposited across the etched area and contacted in the rear by FTO which serves as electrode in a source and drain configuration. The effective light area (0.03cm^2) was defined by the width (0.03cm) of the etched area and the 1 cm gap created at the center of the substrate by placing Kapton tapes on the substrate edges. Photosensing properties were initially measured. Current-voltage (*I–V*) characteristics were investigated using a potentiostat (model M101, Autolab) coupled to a redox.me Switchable LED Light Source and the measurement software. LED Light Source was set at an excitation wavelength of 365 nm and was used to demonstrate the device's photoresponses in the dark and under UV irradiation. The photocurrent measurements were taken by recording the applied direct current (DC) at increasing light power density of; 7, 11, 15, 19 and 23 mW/cm^2, current acquisition interval of 0.02 s, LED switching time of 6 s and a constant applied bias of 1 V using the potentiostat. The light power densities were calibrated using a light power meter (Instytut Fotonowy, Poland). All measurements were conducted at room temperature under ambient conditions.

Table S1. Mean ± standard deviation values of the film thicknesses

Sample	Thickness (nm)
ZnO	486 ± 6
ZnO/□-PVK	740 ± 4
ZnO/δ-PVK	728 ± 8
□-PVK	254 ± 7.2
δ-PVK	242 ± 10

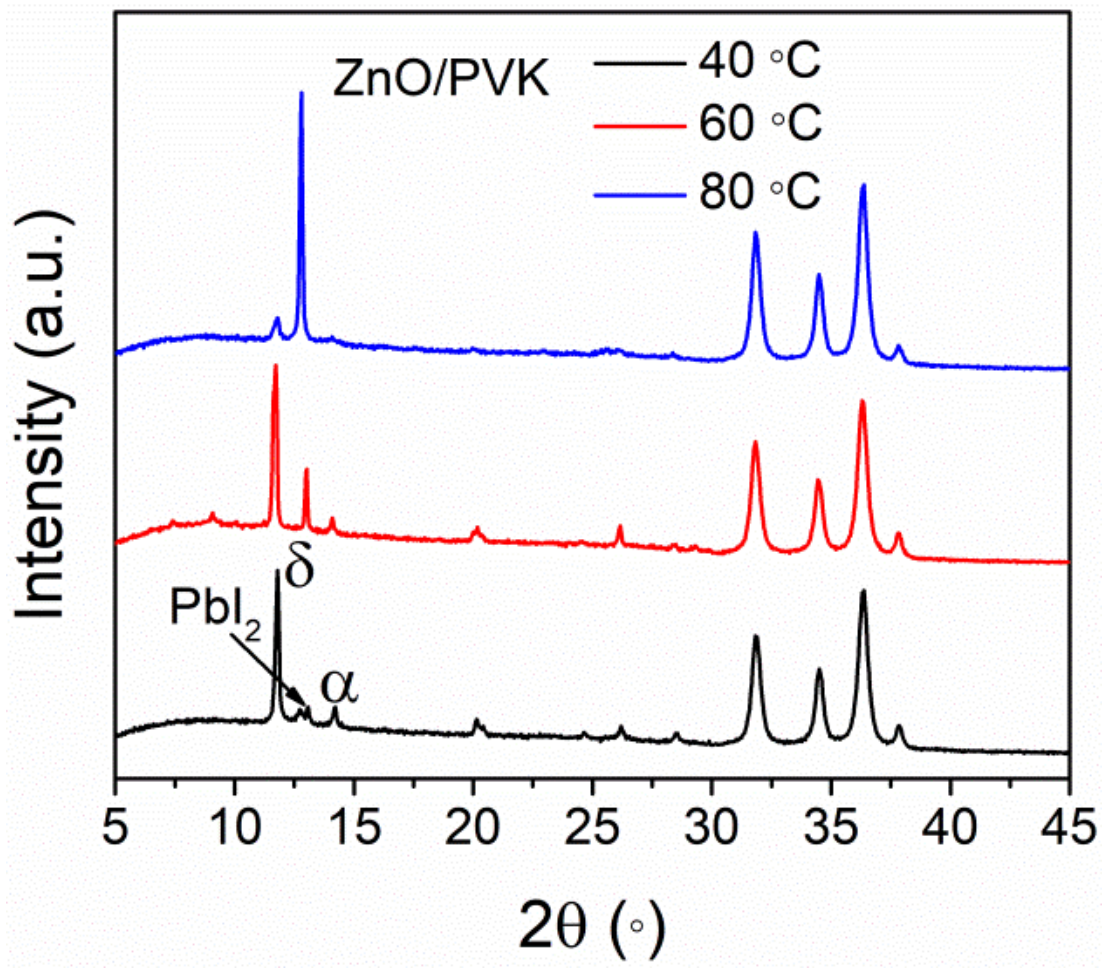

Fig. S1. (a) XRD patterns of ZnO/PVK heterostructure showing further degradation due to prolonged heating at 40 °C, 60 °C and 80 °C.

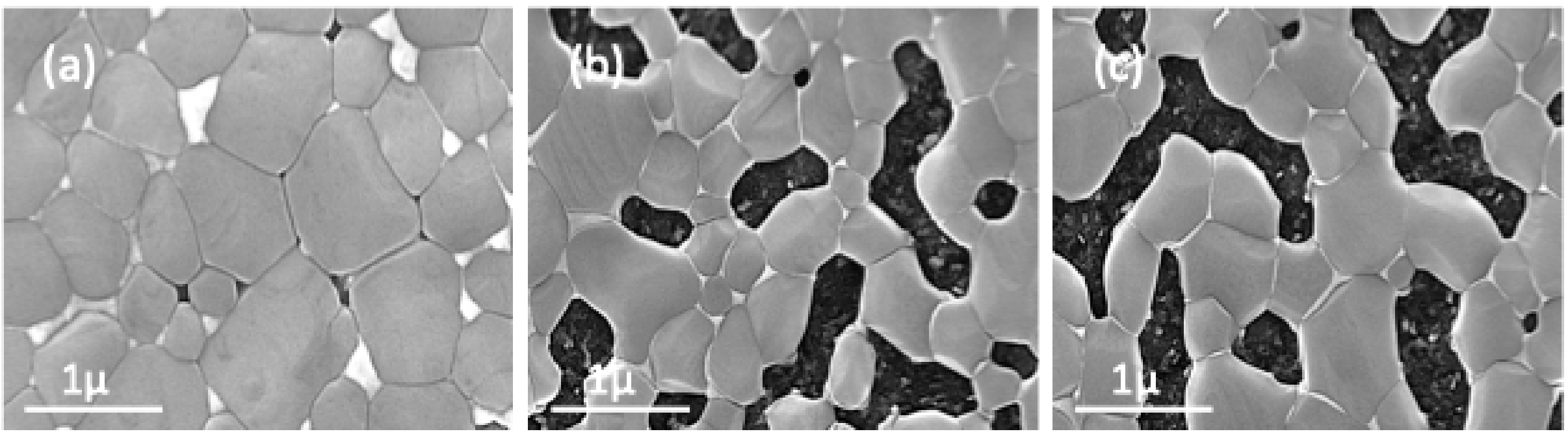

Fig. S2. SEM images of ZnO/PVK with prolonged heating at (a) 40 °C, (b) 60 °C and (c) 80 °C showing intensified surface imperfection i.e. shrinkage of the gains and widening of the void areas with increasing temperature.

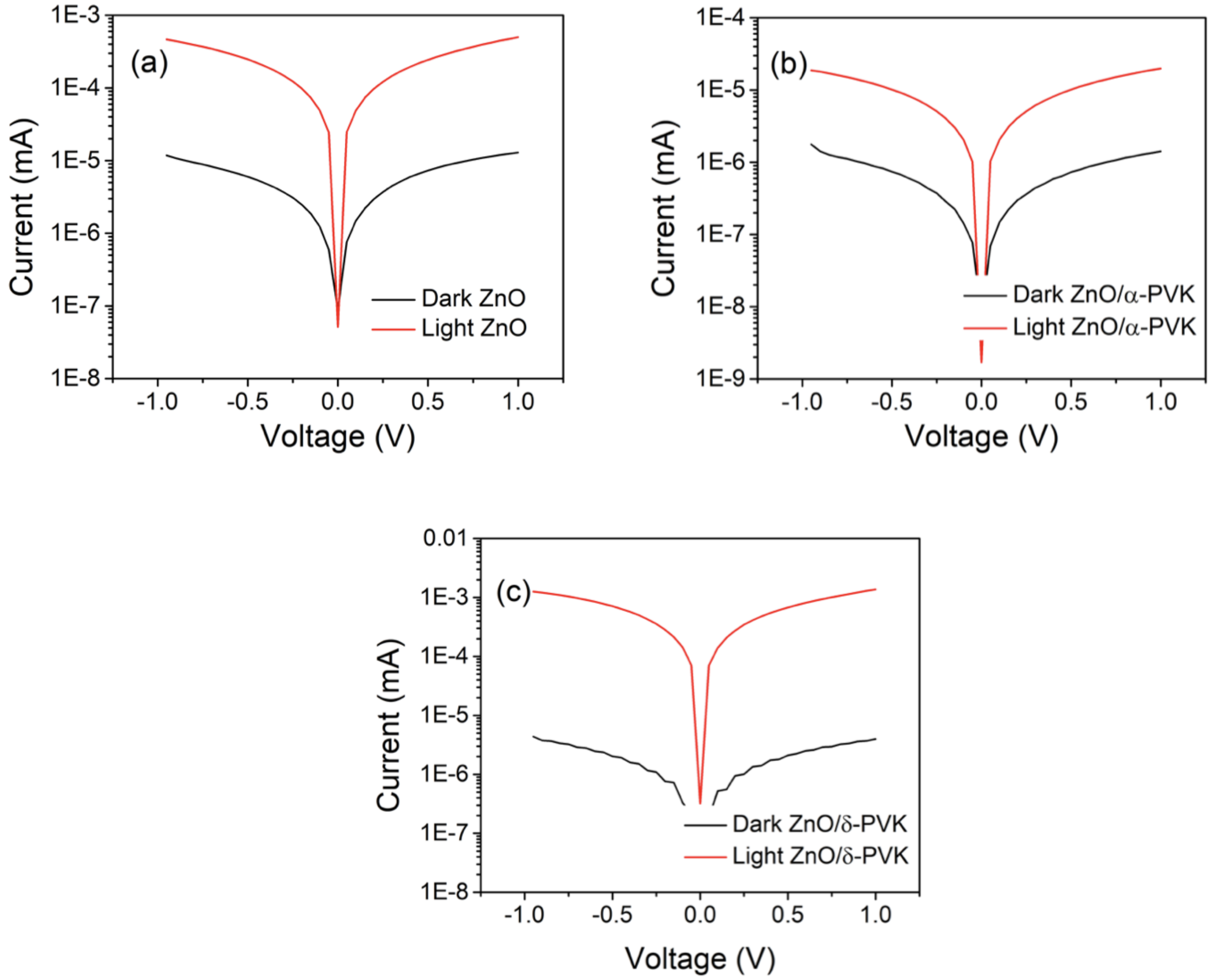

Fig. S3. log plots of the *I-V* characteristics of the devices based on (a) ZnO, (b) ZnO/ □ -PVK and (c) ZnO/δ-PVK in the dark and under 365 nm wavelength of UV light irradiation at 1 V bias.

.

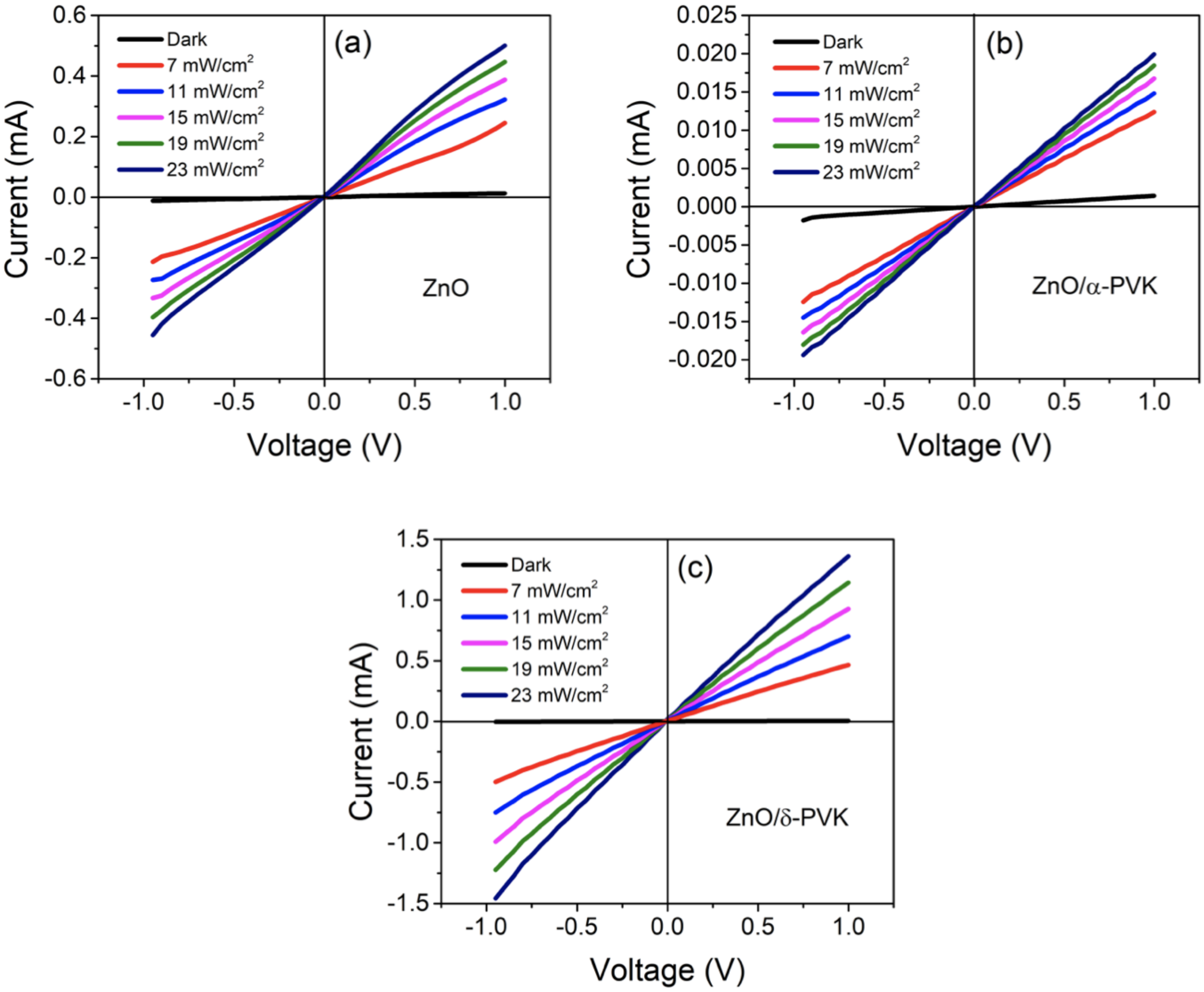

Fig S4. *I-V* characteristics of the devices based on (a) ZnO, (b) ZnO/ □ -PVK and (c) ZnO/δ-PVK in the dark and under UV irradiation showing steady increases in photocurrent with increasing power density (*P*, 7 – 23 mW/cm²) at 1 V bias and 365 nm wavelength of UV light.

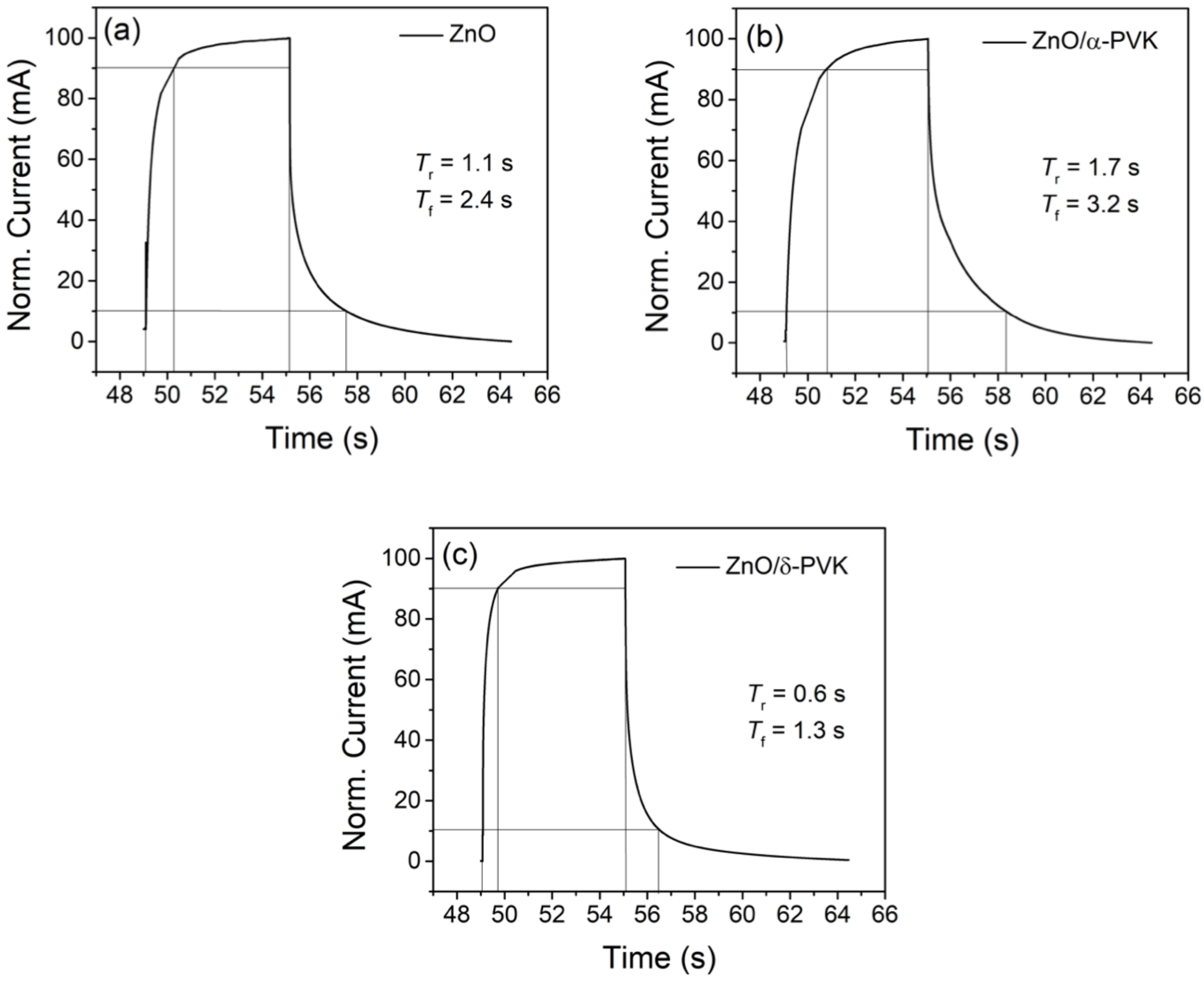

Fig. S5. On/off speed sampled from the normalized response at 23 mW/cm^2 power density with T_r and T_f calculated within the 10 % - 90 % ranges on the "on" and "off" sides of the signals of the devices based on (a) ZnO, (b) ZnO/ □ -PVK and (c) ZnO/δ-PVK.

Table S2. Parameters of devices fabricated in this work and in literature

Active layer	bias (V)	λ(nm)	T_r/T_f (s/s)	I_l/I_d	R (AW^{-1})	D (Jones)	*EQE (%)*	*ref*
ZnO	1	365	1.1/2.4	38.70	0.7	5.96×10^{10}	238	This work
ZnO/□PVK	1	365	1.7/3.2	14.04	0.03	7.70×10^{9}	10	This work
ZnO/δPVK	1	365	0.6/1.3	344.81	2.0	3.06×10^{11}	680	This work
ZnO nanorods	1	375	42.9/132.9	49	0.06	2.12×10^{10}		1
ZnO nanorods	0	365	0.2*		0.0226			2
ZnO nanoparticles	5	365	3.75/3.88	11.12	6.0×10^{-5}	0.62×10^{10}	0.02	3
ZnO nanotube	1	365	9/20	388	4.5×10^{-4}	1.1×10^{10}	0.15	4
ZnO thin film	3.3	375	29.89/7.95		0.023	7.8×10^{10}		5
ZnO nanoclusters	0	375	33.6/36	16.9	3.4×10^{-8}	5.00×10^{9}		6

2D ZnO	1	365	9.4/15.56	5.44	0.06034	4.09×10^{11}	20.45	7
ZnO nanostructure	5	365	5.94/0.77	5783	0.037	1.08×10^{10}		8
ZnO/PVK		360		5.88×10^{4}	2.73	1.09×10^{12}	941	9
ZnO/PVK	0	530	0.02/0.03	610.51	0.47	6.88×10^{11}	110	10
PVK@ZnO	0		0.02/0.02		0.60	1.44×10^{11}		11
PVK/ZnO	5	378			0.1834	1.39×10^{12}	60.2	12
ZnO/PVK	2	350-850	0.081/0.075		0.36	7.80×10^{12}	83.67	13

*Response time

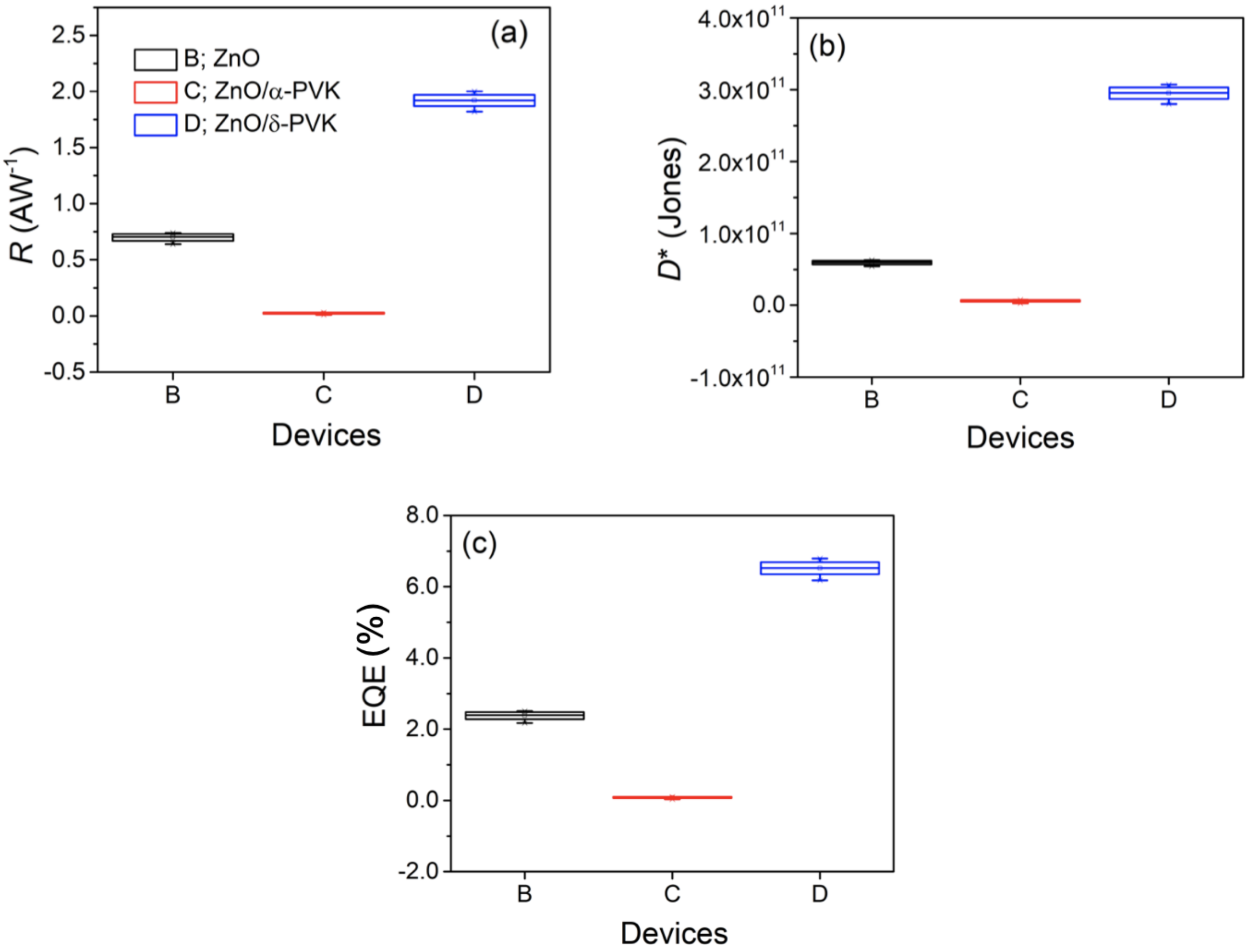

Fig. S6. Box plots of (a) *R,* (b) *D** and (c) EQE obtained from ten independently fabricated devices. The ZnO/δ-PVK devices exhibit the highest median *R, D** and EQE, followed by the pristine ZnO devices while the ZnO/□-PVK devices show the lowest values.

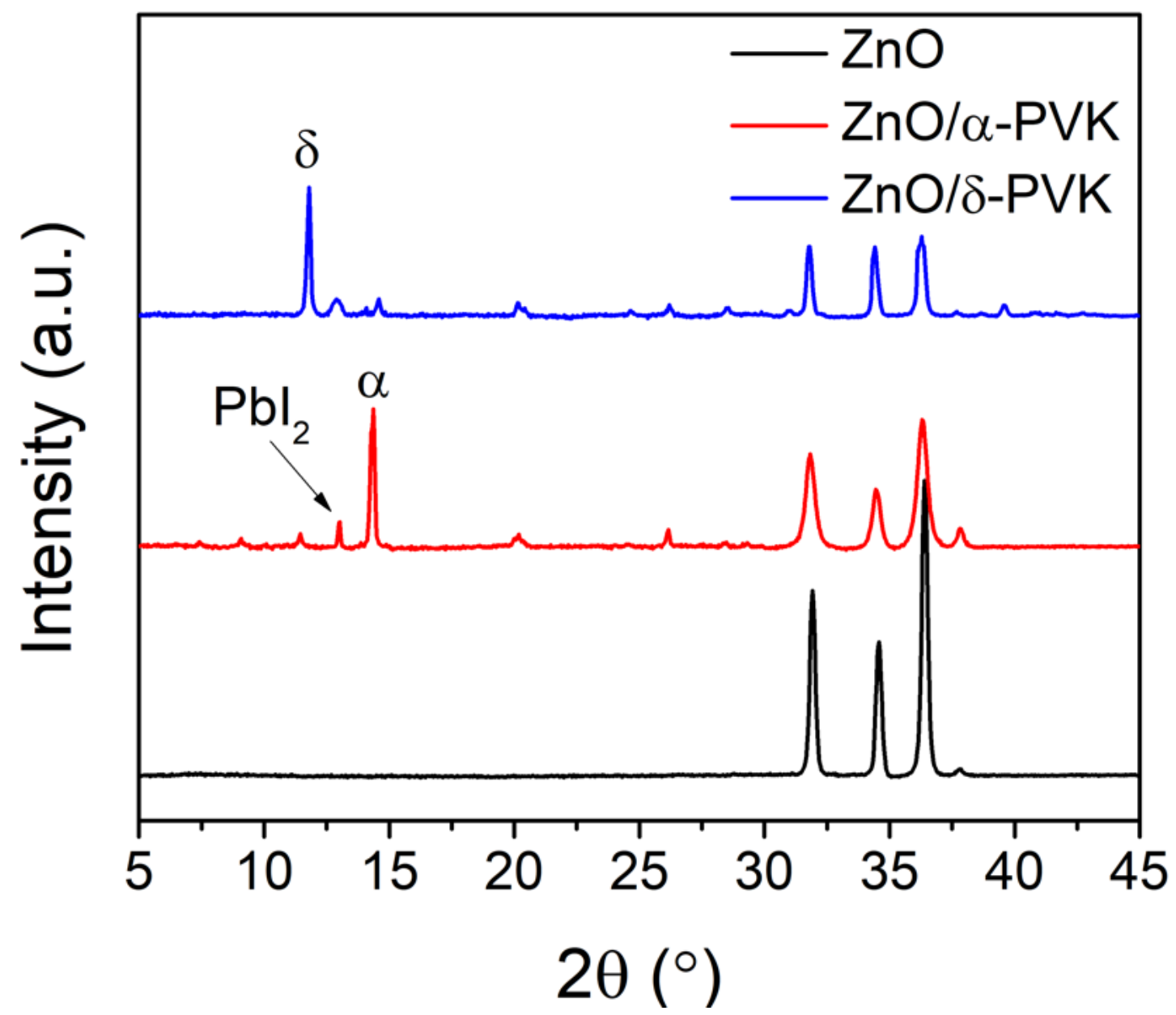

Fig. S7. (a) XRD patterns of ZnO, ZnO/ □ -PVK and ZnO/δ-PVK heterostructures after extended UV irradiation test at 365 nm wavelength, 23 mW/cm² power density and 1 V bias. The ZnO/ □ -PVK heterostructure shows the appearance of a more defined PbI_2 peak and a reduced intensity of the □ peak while the XRD patterns of the ZnO and ZnO/δ-PVK heterostructure remain largely unchanged.

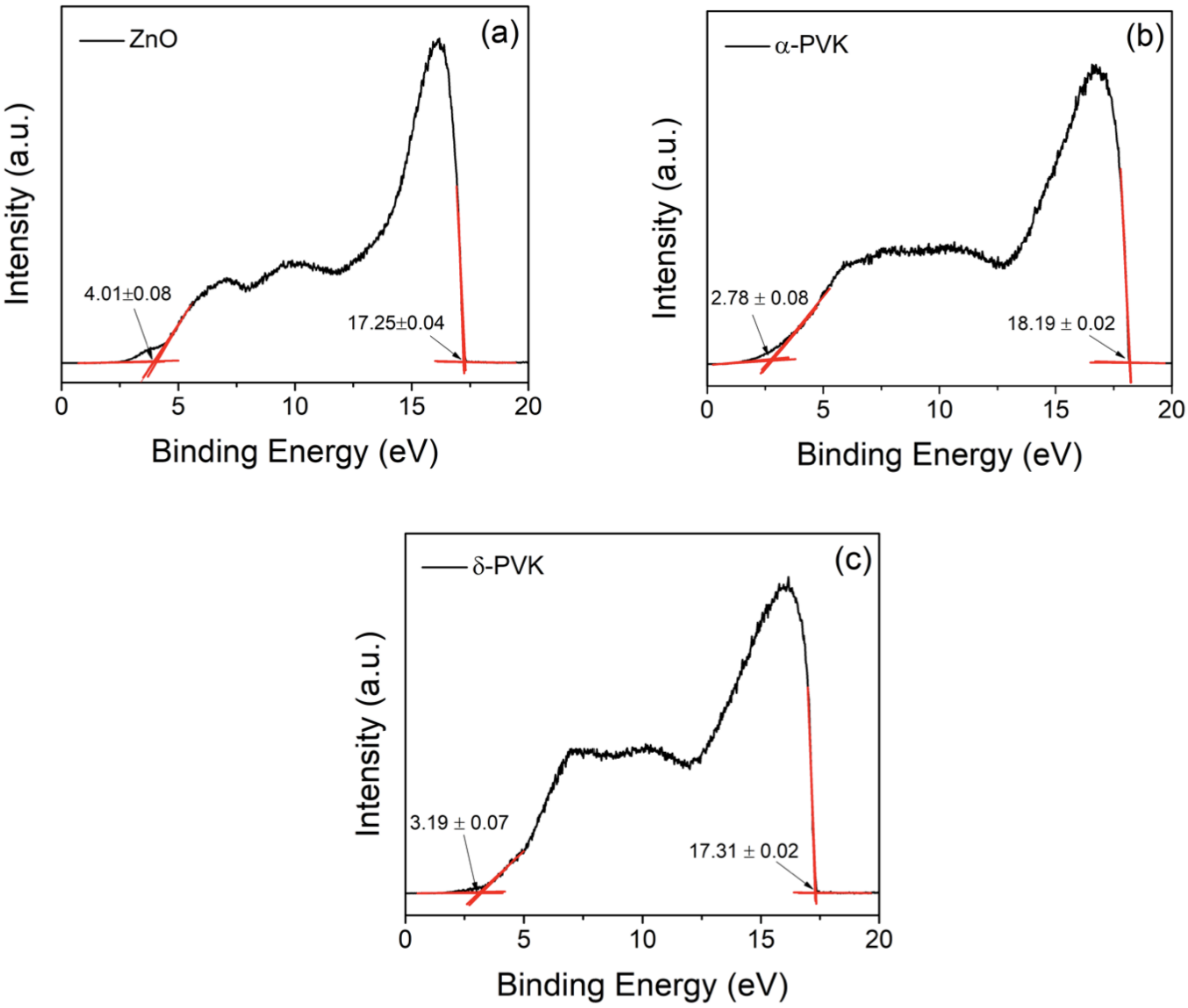

Fig. S8. UPS spectra of (a) ZnO, (b) □-PVK and (c) δ-PVK thin films which were obtained using a helium (He) lamp with an excitation energy of 21.22 eV.

The valence band maximum (VBM) relative to Fermi level (0 eV) and secondary electron cut-off (SECO) onsets were fitted with a linear fitting function:

$$\text{onset; } I_o = m_o \mathrm{E}_{\mathrm{VBM}}^{\mathrm{E_F}} + c_o$$

The VBM relative to Fermi level (0 eV) and SECO baselines were fitted with a linear fitting function:

$$\text{Baseline; } I_b = m_b \mathrm{E}_{\mathrm{VBM}}^{\mathrm{E_F}} + c_b$$

where I_o and I_b are UPS photoemission intensities, m_o and m_b are the slopes of the linear fits and c_o and c_b are the intercepts at the onsets and baselines respectively. $E_{VBM}^{E_F}$ is the valence band maximum relative to Fermi level (0 eV).

$$I_o = I_b \text{ at the intersection}$$

Hence

$$m_o E_{VBM}^{E_F} + c_o = m_b E_{VBM}^{E_F} + c_b$$

$$E_{VBM}^{E_F} = \frac{c_b - c_o}{m_o - m_b}$$

To obtain reliable values, each fitting was done 3 times to achieve 3 different fitting windows (denoted as W1, W2 and W3 in Table S3). The mean values and standard deviations of the VBM and SECO were obtained and the actual VBM (E_{VBM}) values were calculated using the mean values and the excitation energy of 21.22 eV from helium (He) lamp based on the equation

$$E_{VBM} = -(WF + E_{VBM}^{E_F})$$

Where WF (work function) = 21.22 - SECO

Table S3. Fitting parameters to obtain E_{VBM} for ZnO, □ -PVK and δ-PVK thin films

Thin film		W1		W2		W3	
**ZnO**	VBM onset	m_o	10907	m_o	12227	m_o	10833
		c_o	-42630	c_o	-49461	c_o	-42522
	Baseline	m_b	197	m_b	253	m_b	115
		c_b	-216	c_b	-332	c_b	-95
		$E_{VBM}^{E_F}$	3.96	$E_{VBM}^{E_F}$	4.10	$E_{VBM}^{E_F}$	3.96
	$E_{VBM}^{E_F}$				4.01 ± 0.08		
	SECO onset	m_o	-147496	m_o	-199496	m_o	-218032
		c_o	2550850	c_o	3438140	c_o	3754390
	Baseline	m_b	-179	m_b	-109	m_b	-109
		c_b	3471	c_b	2205	c_b	2168
		E_{SECO}	17.30	E_{SECO}	17.23	E_{SECO}	17.22
	E_{SECO}				17.25 ± 0.04		

	E_{VBM}			-7.98 eV			
□ -PVK	VBM onset	m_o	18143	m_o	16704	m_o	17719
		c_o	-47655	c_o	-42796	c_o	-47419
	Baseline	m_b	1418	m_b	953	m_b	1321
		c_b	-712	c_b	-470	c_b	-859
		$E_{VBM}^{E_F}$	2.81	$E_{VBM}^{E_F}$	2.69	$E_{VBM}^{E_F}$	2.84
	$E_{VBM}^{E_F}$			2.78 ± 0.08			
	SECO onset	m_o	-323962	m_o	-301741	m_o	-331539
		c_o	5891560	c_o	5495080	c_o	6031960
	Baseline	m_b	-136	m_b	-186	m_b	-398
		c_b	3292	c_b	4260	c_b	8336
		E_{SECO}	18.17	E_{SECO}	18.21	E_{SECO}	18.19
	E_{SECO}			18.19 ± 0.02			
	E_{VBM}			-5.81 eV			
δ-PVK	VBM onset	m_o	10454	m_o	11314	m_o	11152
		c_o	-31943	c_o	-35738	c_o	-35254
		m_b	87	m_b	249	m_b	12
		c_b	355	c_b	191	c_b	419
		$E_{VBM}^{E_F}$	3.12	$E_{VBM}^{E_F}$	3.25	$E_{VBM}^{E_F}$	3.20
	$E_{VBM}^{E_F}$			3.19 ± 0.07			
	SECO onset	m_o	-284613	m_o	-307573	m_o	-334877
		c_o	4932010	c_o	5323760	c_o	5791790
	Baseline	m_b	-80	m_b	-94	m_b	60
		c_b	2065	c_b	2302	c_b	-562
		E_{SECO}	17.33	E_{SECO}	17.31	E_{SECO}	17.29

E_{SECO}	17.31 ± 0.02
E_{VBM}	**7.10 eV**

References

1. S.-J. Chang, C.-W. Liu, C.-H. Hsiao, K.-Y. Lo, S.-J. Young, T.-H. Kao, K.-S. Tsai, S.-L. Wu, *IEEE Photon. Technol. Lett.,* 2013, **25,** 2089-2092.

2. M.-Y. Lu, H.-Y. Chen, C.-Y. Tsai, Y.-T. Tseng, Y.-T. Kuo, H.-C. Wang, M.-P. Lu, *Chem. Commun.,* 2016, **52,** 12853-12856.

3. C. Gautam, A. Verma, P. Chaudhary, B. C. Yadav, *Opt. Mater.,* 2022, **123,** 111860.

4. M. Zhou. B. Wu, X. Zhang, S. Cao, P. Ma, K. Wang, Z. Fan, M. Su, *ACS Appl. Mater. Interfaces,* 2020, **12,** 38490-38498.

5. X. Chen, S. Huang, N. Nasiri, *ACS Appl. Nano Mater.,* 2024, **7,** 3589-3600.

6. X. Chen, B. P. Veettil, N. Nasiri, *Nano Today,* 2025, **63,** 102754.

7. A. Verma, P. Chaudhary, R. K. Tripathi, B. C. Yadav, *Sens. Actuators A: Phys.,* 2021, **321,** 112600.

8. J. Wang, C. Chen, M. Jin, Y. Zhu, N. Zhang, *Opt. Mater.,* 2023, **135,** 113341.

9. Y. Peng, D. Jiang, M. Zhao, Y. Duan, H. Wei, H. Li, Q. Liang, S. Wang, *J. Alloys Compd.*, 2023, **965,** 171372.

10. L. Chu, C. Xu, D. Liu, C. Nie, X. Zhou, L. Deng, *Mater. Sci. Semicond. Process.*, 2024, **169,** 107943.

11. Q. Wang, Y. Li, X. Cao, G. Li, Y. Zhao, X. Liu, H. Cao, X. Zhang, F. Chen, Y. Yan, F. Liu, Y. Ma, L. Yang, *J. Alloys Compd.*, 2025, **1032,** 181145.

12. D. Shao, W. Zhu, G. Xin, X. Liu, T. Wang, S. Shi, J. Lian, S. Sawyer, *J. Mater. Chem. C*, 2020, **8,** 1819-1825.

13. R. K. Upadhyay and S. Jit, *Opt. Mater.*, 2023, **135,** 113290.
